# Study of Medical Ultrasound for Rhizarthrosis (SUR): study protocol for a randomized controlled single-center pilot-trial

**DOI:** 10.1186/s13063-020-04375-2

**Published:** 2020-06-01

**Authors:** Markus Bock, Andreas Eisenschenk, Heiko Lorenzen, Martin Lautenbach

**Affiliations:** 1 Department of Hand Surgery, Upper Extremity and Foot Surgery, Center for Orthopedics and Trauma Surgery, Hospital Waldfriede, Argentinische Allee 40, 14163 Berlin, Germany; 2 Institute of Biochemistry, University Medicine Berlin – Charité, Charitéplatz 1, 10117 Berlin, Germany; 3 Department of Neurology, University Medical Center of the Johannes-Gutenberg University Mainz, Langenbeckstr. 1, 55131 Mainz, Germany; 4 Department of Hand and Functional Microsurgery, University Medicine Greifswald, Ferdinand-Sauerbruch-Str. 1, 17475 Greifswald, Germany; 5 Department of Hand, Replantation and Microsurgery, Unfallkrankenhaus Berlin, Warener Str. 7, 12683 Berlin, Germany; 6 Practice for Occupational Therapy, Argentinische Allee 40, 14163 Berlin, Germany

**Keywords:** Degeneration, Rhizarthrosis, Trapeziometacarpal osteoarthritis, Thumb basal joint arthritis, Thumb carpometacarpal joint arthritis, Hand therapy, Therapeutic ultrasound

## Abstract

**Background:**

Rhizarthrosis (trapeziometacarpal osteoarthritis) is the second most common site of osteoarthritis in the hand affecting 10–30% of adults over the age of 50. Up to four times as many women as men have rhizarthrosis. Clinical symptoms include functional disability of the thumb, pain, joint swelling, and reduced strength. The first carpometacarpal joint is pivotal in the opposition of the thumb and allows a high degree in flexibility to humans. Current therapies focus mainly on surgical strategies, which should be considered in advanced, therapy-resistant stages to relieve pain and improve function. However, conservative treatment methods are urgently required in presurgical stages. The efficacy of conservative treatment options for rhizarthrosis, which are intended to preserve function, joint integrity and to relieve pain, has not been adequately studied. In the clinical study protocol presented here, we investigate the efficacy of multimodal hand therapy versus therapeutic ultrasound versus combination therapy with both hand therapy and therapeutic ultrasound.

**Methods:**

This study is a single-center, randomized, controlled, parallel-group pilot trial. One hundred fifty patients with rhizarthrosis and current disease activity will be randomized to one of three conservative interventions over 6 months. Interventions are (1) multimodal hand therapy (2) therapeutic ultrasound, and (3) combination therapy with both hand therapy and ultrasound therapy. The primary outcome measure is the Disabilities of the Arm, Shoulder, and Hand (DASH) questionnaire score after 6 months. Secondary endpoints are changes in pain, quality of life, disability progression, and changes of hand function. Safety will also be assessed.

**Discussion:**

Clinical data suggest that multimodal hand therapy may improve functionality and reduce pain in rhizarthrosis. Clinical data regarding therapeutic ultrasound are not available. Clinical evidence is lacking. This study is the first clinical study investigating the effects of multimodal hand therapy in direct comparison to therapeutic ultrasound and to a combination therapy with both hand therapy and ultrasound therapy for rhizarthrosis.

**Trial Registration:**

ClinicalTrials.gov; NCT04115085; Registered on September 30, 2019.

## Introduction

Rhizarthrosis (or osteoarthritis of the trapeziometacarpal joint) (OAT) is a common form of osteoarthritis in the hand leading to chronic disability in humans over 50. Although the exact mechanisms of osteoarthritis are still elusive, various person-level risk factors are recognized [1–9]. Although the hand X-ray is the gold standard for diagnosing and monitoring OAT, it has limited correlation to clinical symptoms [2, 10, 11]. The prevalence ranges between 4 and 36% depending on the diagnostic criteria used, rises sharply at ages over 60, and is thought to be twice to four times higher in women [2, 9–13] . OAT leads to articular cartilage damage, adjacent tissue degeneration, and may result in total joint destruction. Moreover, OAT is a major cause of progressive disability, early retirement, and depression in adults [8, 14, 15]. Currently, there is no cure for OAT, but several surgical therapies are available, with or without alloplastic reconstruction, which may improve function und reduce pain [16, 17]. However, they all may have substantial side effects, failure rates, and patients respond differently due to the complex nature of the disease. Therefore, conservative options should be considered first [18]. Consequently, there is a need for conservative therapies such as specific hand therapies that may reduce OAT symptoms, improve the patient’s quality of life, and, at best, delay disease progression [19]. It is unclear which conservative measures, if any, are most effective [20]. The aim of conservative treatment is to restore thumb functionality, including pain relief, stability, motility, and strength. Treatments commonly used prior to surgery include injections (cortisone, hyaluronate), analgesics, patient education in joint protection, strengthening exercises, assistive devices, and orthotics [21, 22]. Few randomized clinical trials on conservative treatment of OAT have been published [22–27]. There is increasing evidence favoring multimodal manual treatment to reduce pain and ameliorate function in OAT patients [26, 28–30]. For more than 60 years, therapeutic ultrasound (TUS) has been a non-invasive method to treat musculoskeletal pain, soft tissue injuries, and chronic wounds with debatable efficacy [31, 32]. However, recently TUS was reported to improve function, tissue regeneration, and relieve pain via thermal and non-thermal mechanisms in osteoarthritis of the knee [33–36]. Experimental data suggest that TUS treatment might prevent degenerative changes of the meniscus and exert a reparative effect via activation of cartilage growth factors [37]. Despite such evidence of efficacy, to the best of our knowledge, TUS has never been studied in OAT. Despite this lack of proven efficacy, based on our clinical experience, physiotherapists frequently use TUS to treat rhizarthrosis. It is of interest that TUS was used as a harmless, non-invasive ‘sham procedure’ in nontherapeutic doses in several clinical studies in OAT [20]. The aim of the present study is (1) to provide a direct comparison of the efficacy of two promising and readily available conservative treatment methods for symptomatic OAT, namely multimodal hand (MHT) therapy and TUS, (2) to suggest treatment recommendations, and (3) to offer direction for future studies. This research is expected to have a positive impact on OAT patients, because if MHT or TUS can reduce pain and improve function of the basal thumb joint, the significant disease burdens associated with pain and psycho-emotional stress, lost productivity, and frequent health care visits will also be reduced.

## Methods

### Study design

**Fig. 1:**
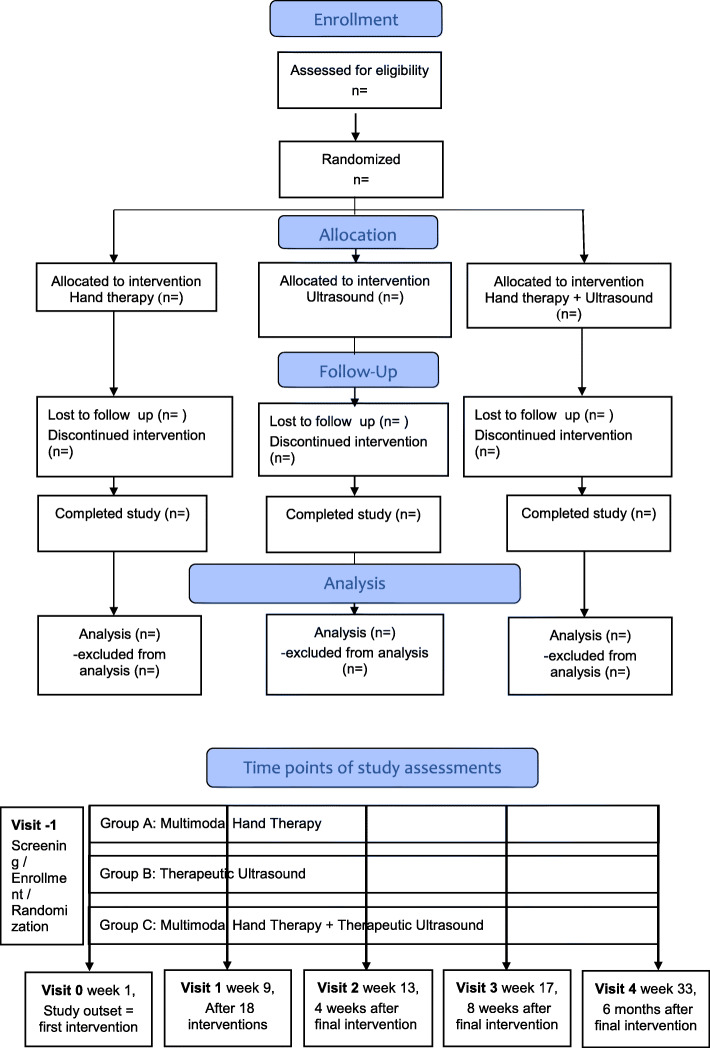
SUR study flow chart. *SUR* Study of Medical Ultrasound for Rhizarthrosis

This is a single-center, randomized, controlled, repeated-measures, three-armed, parallel-group study conducted at the Waldfriede Hospital Berlin. Recruitment started in October 2019 (ClinicalTrials.gov identifier: NCT04115085). Patients are recruited from our hand surgery outpatient clinic on the Waldfriede Hospital campus. Further recruitment strategies include specific study calls on the website of the Waldfriede Hospital and information events and lectures for patients including distribution of study flyers. We intend to randomize 150 OAT patients to one of three hand therapeutic interventions. Intervention groups are (A) MHT with 18 sessions over 9 weeks, and (B) therapeutic TUS with 18 sessions over 9 weeks, and (C) combination therapy with MHT directly followed by TUS with 18 sessions over 9 weeks. Participants are studied on the first day before the intervention starts and at 9, 13, 17 and 33 weeks after the start of the intervention. The institutional review board of Waldfriede Hospital Berlin gave a positive evaluation for the study and written informed consent was obtained from all participants prior to study entry (Additional files 1 and 2). The study is conducted in accordance with the Declaration of Helsinki in its currently applicable version, the guidelines of the International Conference on Harmonization of Good Clinical Practice (ICH-GCP) and applicable German laws. Table 1 and Fig. 1 show the visit schedule and the study flow chart, respectively. Participants do not receive remuneration. For further details refer to the SPIRIT checklist (Additional file 2).

**Table 1:** SPIRIT SUR study schedule

Visit	-1 Screening	0 Baseline Start intervention	1 End of intervention	2 Follow-Up	3 Follow-Up	4 Final follow-up visit
Week	−12 (max)	1	9	13	17	33
Informed consent	x					
Demographics	x					
Inclusion / exclusion criteria	x	x	x	x	x	x
Case history	x					
Medication	x	x	x	x	x	x
DASH		x	x	x	x	x
SF-36		x	x	x	x	x
VAS pain		x	x	x	x	x
Key pinch force		x	x	x	x	x
Goniometry		x	x	x	x	x
MHT		x	x			
TUS		x	x			
MHT + TUS		x	x			
Physical examination	x				x	
AE / SAE query		x	x	x	x	x

*AE* adverse event, *DASH* Disabilities of the Arm, Shoulder, and Hand, *MHT* multimodal hand therapy, *SAE* serious adverse event, *SF-36* Short Form 36, *SPIRIT* Standard Protocol Items: Recommendations for Interventional Trials, *SUR* Study of Medical Ultrasound for Rhizarthrosis, *TUS* therapeutic ultrasound, *VAS* visual analogue scale

### Participants

Patient information and informed consent have been prepared in accordance with the guidelines of the institutional review board of Waldfriede Hospital Berlin. Potential participants receive both forms at least 24 hours before one of the study physicians personally explains all study procedures to them. If he or she is willing to participate and has had sufficient time to ask questions, written informed consent is given. Afterwards, inclusion and exclusion criteria are assessed. Main inclusion criteria are a definite diagnosis of OAT according to the stage I to IV Eaton and Littler X-ray criteria [38] and clinical symptoms in the region of the thumb base for at least 3 months.

#### Complete list of inclusion criteria

- Male and female patients aged 40 to 90 years
- Pain intensity in the region of the thumb base in pain phases ≥40 mm on the visual analogue scale (VAS) of 0 to 100 mm (relative to the last 24 hours)
- Complaints for at least 3 months
- X-ray Eaton and Littler stage I–IV determined at least once
- Ability to give verbal and written consent
- Health insurance

#### Complete list of exclusion criteria

- History of surgery on the affected hand, wrist, or forearm
- Planned surgery within the next 8 months
- Anticoagulation (Vitamin-K antagonists, heparin)
- Hemophilia, von Willebrand-Jürgens syndrome, thrombocytopathy, or other blood anomalies
- Acute pain medication (< 7 days)
- Systemic medication with corticoids or immunosuppressants
- Intra-articular injections or Radiosynoviorthesis (radiosynovectomy) (RSO) within the last 3 months
- Pregnancy, lactation
- Significant cognitive impairment
- Clinically relevant or progressive systemic disease (e.g., liver, kidney, cardiovascular system, respiratory tract, vascular system, brain, metabolism, thyroid) that could affect the course of the study
- Malignant disease
- Simultaneous participation in an interventional study or participation in an interventional study in the last 2 months before study inclusion
- Clinically relevant addiction or substance abuse disorder (defined as alcohol or drug abuse)
- Insufficient mental capacity to cooperate
- Suspected lack of compliance
- Medical, psychiatric or other conditions that restrict the patient’s following abilities: to interpret the study information, to give informed consent, to adhere to the rules of the protocol, or to complete the study

Discontinuation criteria are withdrawal of consent, subsequent occurrence of an exclusion criterion, lack of compliance, and medical reasons for stopping the intervention. Participants meeting a discontinuation criterion are offered the chance to attend the remaining study visits for follow-up outside the study protocol.

### Randomization

After written informed consent is obtained, patients eligible for the study are randomly allocated to either group A, B, or C using a permuted block randomization scheme generated by an external statistician not involved in the study. We use a block randomization with variable block length. The varied block sizes were chosen to prevent predictability in treatment allocation. A clinician not involved in this trial keeps the randomization list and, when called, announces the intervention group to which the participant has been assigned.

### Blinding

This is an open-label study. Analysts who evaluate the primary endpoint are blinded for clinical data and interventional allocation. Hand therapists are blinded for patient-reported and disease-related data.

### Conservative interventions

*Group A: Multimodal Hand Therapy as introduced by Kaltenborn and exercise at home* [39, 40].

Over a period of 9 weeks, MHT is applied twice per week to the first carpometacarpal joint on the symptomatic side or bilaterally for 20 minutes or 40 minutes, respectively per session. The therapy is conducted by an experienced hand therapist.

In all cases, the therapy session is performed as follows:

1. *Manual therapy:* Transverse friction massage of the thenar muscles with moderate intensity applied two times followed by a 1-minute rest period.
2. *Manual muscle release:* Repetitive dorsal stretching of the thenar muscles for 3 minutes followed by a 1-minute rest period.
3. *Manual therapy*: Traction technique for grade II and III pain relief three times for 1 minute, followed by a 1-minute rest.
4. *Strengthening exercise:* The carpometacarpal 1 (CMC-1) joint is in palmar abduction, the metacarpophalangeal 1 (MCP-1) and interphalangeal (IP) joints in flexion. The patient is asked to sustain this position of thumb opposition to the index fingertip of the same hand. The patient is asked to repetitively extend the index finger by maintaining the position of the thumb and bring the index finger back in opposition, meeting the tip of the thumb three times for 1 minute, each time followed by a 1-minute rest period.
5. *Strengthening exercise:* The CMC-1 joint is in radial abduction and moderate extension and the patient is asked to sustain that position. Next, alternate abduction and adduction of the index finger is performed for strengthening the first dorsal interosseous muscle by moving an elastic band for 2 minutes.
6. *Home strengthening exercise:* All participants must additionally perform a daily home exercise program. The two exercises are explained and an exercise pictogram is provided. The exercises are identical to paragraphs number 4 and 5 above.

*Group B: Application of non-pulsed and pulsed therapeutic ultrasound.*

Over a period of 9 weeks, TUS is applied to each affected CMC 1 joint for 10 minutes twice per week. There are 18 therapeutic units per affected hand in total. The patient sits in front of the assessment table; forearm and hand lie in dorsal position on the table. For the first 5 minutes, TUS is applied with a hand-held transducer in a continuous (non-pulsed) wave mode with an intensity of 1 W/cm^2^. Immediately after this procedure, the same hand-held transducer is used to apply the pulsed wave ultrasound with an intensity of 0.3 W/cm^2^ at a pulse repetition frequency of 100 Hz, and a duty cycle of 20% (ratio 1:5) for 5 minutes. Both modes (pulsed and non-pulsed ultrasound) deliver ultrasonic energy at a frequency of 2.4 MHz. Due to likely enhanced positive effects of pulsed TUS [35], a subgroup of 15 randomly assigned patients from group B receive pulsed TUS for 10 minutes and do not receive continuous TUS. Thus, the same transducer will be used and ultrasonic energy at a frequency of 2.4 MHz, average temporal intensity of 0.3 W/cm^2^ at a pulse repetition frequency of 100 Hz, and a duty cycle of 20% (ratio 1:5). All TUS treatments are conducted by experienced hand therapists and the transducer is applied with coupling gel and moved in circular motion over the CMC 1 joint. The operator, the device, the contact gel, and the position of the patients are the same at all visits. We use the ultrasound device Modell Soleo Sono (Zimmer MedizinSysteme GmbH, Neu-Ulm, Germany) and the ultrasonic transducer US S SD/SL, diameter 13 mm (1cm^2^) and an effective radiating area (ERA) of 0.65cm^2^ at 2.4 MHz.

### Adverse events

Patients will be asked about the tolerability of the interventions and any adverse events (AEs) will be recorded. We do not expect serious adverse events (SAEs)due to our non-invasive and harmless interventions. There is no anticipated harm for trial participation and thus no provisions for post-trial care or compensation.

### Outcome parameters

Primary outcome is the change in the disease-specific Disabilities of the Arm, Shoulder, and Hand (DASH) questionnaire score after 9, 13, 17, and 33 weeks compared to baseline [41, 42].

Secondary patient-reported outcomes include: (1) VAS pain determined by change in distance between the patient-made mark after 9, 13, 17, and 33 weeks compared to baseline; (2) Short Form 36 (SF-36) determined by scale score change after 9, 13, 17, and 33 weeks compared to baseline [43]. Secondary disease-related outcomes include: (3) Thumb force or key pinch force evaluation, determined by change in Kg after 9, 13, 17, and 33 weeks compared to baseline. All thumb force evaluations will be assessed in the following position: Thumb on top of the radial side of the index finger below. (4) Goniometry measurement determined by change in range of motion after 9, 13, 17, and 33 weeks compared to baseline. All goniometric evaluations assess the active range of motion (ROM) of the angle between the dorsal axis of the thumb and the index finger with a manual goniometer.

All clinical assessments will be performed by the same experienced evaluator at all time intervals, who is blinded to the treatment allocation.

For safety monitoring, patients will be asked for tolerability of the interventions and in case of AEs a medical doctor is always at our site to provide help. For a detailed overview of assessments and endpoints see Table 1.

### Power calculation

Since this study is planned as a pilot study, formal statistical power calculations are not performed for all measures and tests of interest. The primary limitation for making power calculations is the lack of available information on what would constitute a clinically significant effect size for the measures of interest in OAT. Thus, we refer to other studies (regarding osteoarthritis of the knee and ultrasound therapy) for a “sample size calculation” since there is no justification with regard to statistical power [34, 35, 44]. The parameters determined with this pilot study will be used to plan the number of cases in a subsequent controlled prospective randomized trial. However, we estimated a sample size of 45 patients per group and defined a two-sided significance level of 0.05 for all tests. We expect a dropout rate of approximately 10% and therefore plan to enroll 50 patients per group, or a total of 150 patients.

### Data management

Each participant will be assigned a unique identifier upon study entry. This identifier will be used for all data documentation to assure the participant’s confidentiality. Data will be collected in source documents and then transferred onto paper case report forms. Later, all data will be digitized and stored in a central database. To improve the accuracy of data entry, entries will be verified for proper format and expected range as well as double-checked. Overall data quality will be assured by an independent monitor throughout the study. However, due to the minimal risks of our MHT and TUS interventions, an interim analysis or formal plan to stop the trial are deemed unnecessary. In terms of data and safety monitoring, a Safety Officer (SO) will be designated. The SO has no financial, scientific, or other conflict of interest with the trial. The SO meets with the Primary Investigator (PI) twice annually to review study progress, data quality, and participants’ safety. Data will be stored for 10 years after study completion and then deleted. Any modification to the current study protocol will be submitted to the institutional review board, to all trial participants, and to the trial investigators. On the consent form, participants will be asked if they agree to use of their data should they choose to withdraw from the trial. Participants will also be asked for permission for the research team to share relevant data with people from the universities taking part in the research or from regulatory authorities, where relevant. This trial does not involve collecting biological specimens for storage. Results will be explained to all study participants individually, presented at national and international conferences, published in peer-reviewed journals, and disseminated to surgeons, physio/hand therapists and the medical laity. We will comply with the official eligibility guidelines for authorship for all publications and do not intend to use professional writers.

### Data analysis

The data collected are described using statistical parameters. Continuous data are described with mean, standard deviation, minimum, maximum and 95% confidence intervals; for categorical data, the frequencies attained and the associated percentages are given with 95% confidence intervals.

The statistical analysis will only include the patients who complete the study in accordance with the protocol. Sub-collectives can be examined exploratively if this makes sense after the survey has been completed. In addition to the primary analysis of the comparison of patients with and without ultrasound therapy, there is a comparison of the patients between the different groups.

Group differences (MHT versus TUS versus study group with MHT + TUS) regarding the primary endpoint are analyzed with the non-parametric Kruskal-Wallis test for independent samples. After the global test, the groups are compared in pairs (Mann-Whitney tests). Secondary endpoints are evaluated according to the scaling of the data but are also always tested non-parametrically. The evaluation of intra-group differences (DASH or other characteristics after 9, 13, 17, and 33 weeks) is carried out by means of a nonparametric analysis of variance for repeated observations (Friedman test). In line with the nature of the pilot study, no adjustment is made for multiple testing. A *p* < 0.05 is considered (in the exploratory sense) to be statistically significant. Missing values will be replaced using multiple imputation [45]. All occurring AEs (AEs and SAEs) are described individually.

## Discussion

Rhizarthrosis is a common and burdensome condition that causes substantial morbidity, disability, frequent ambulant health care visits, and hospitalization [5, 14]. Conservative therapies are needed because prevalence rates for osteoarthritis are rising and conservative treatment options have been scientifically underexplored [6, 19, 46]. Therapeutic exercise and manual hand therapy and their combination have been reported as effective methods to improve pain and function in short-term follow-up in OAT patients [20, 29, 47]. However, there is a body of evidence that TUS is an upcoming, highly effective, non-invasive, and time-sparing therapy in osteoarthritis. Recently, TUS was reported to improve pain, regeneration, and function in osteoarthritis of the knee [33–37, 48]. Despite these reports, no one, to the best of our knowledge, has studied TUS in OAT patients. To date, ours is the first study that investigates the efficacy of a 9-week intervention using MHT and TUS on patient-reported and disease-related measures in OAT patients, including a long-term period of follow-up over 6 months. We expect the findings from this trial to lead to new insights into the effectiveness and sustainability of manual therapy, ultrasound therapy, and a combination of both. OAT has been associated with more severe upper extremity disabilities as determined by DASH scores [49]. Consequently, we have chosen the DASH instrument as the primary outcome. In addition, we will assess objective measures, such as key pinch force and other disease-related and patient-reported outcomes, such as pain, health-related quality of life, and range of motion. There are several reasons for the lack of such large-scale, long-term studies. The predominant reason, of course, is the high demand of financial and human resources. Further, patients often prefer one particular intervention, and are thus apt to withdraw consent when randomized to an undesired intervention.

There are some limitations of our study design. First, a rehabilitative intervention study cannot be completely blinded. Thus, expectations and observer bias cannot be ruled out. However, we try to minimize bias by not communicating any longitudinal data during the study to patients and study personnel in contact with them. More important, the hand therapists who are implementing all of the interventions are blinded for both patient-reported and disease-related data. An additional limitation is the lack of formal power calculation due to the characteristics of an exploratory study.

One great strength of our study is that it can fill the treatment gap in osteoarthritis care, for many patients do not currently receive recommended conservative treatment before being referred for orthopedic surgery [50].

Further strengths of the study are the randomized study design, blinded outcome assessment, large sample size, long intervention of 18 treatment sessions, and 6 months of follow-up. Moreover, we focus on disease-related outcomes, but also on several patient-related outcomes, such as thumb force, thumb range of motion, pain scale and quality of life.

In conclusion, MHT and TUS are potentially safe, cost- and time-effective treatment options for OAT, and our study might close the gap between uncertainty of doctors and manual therapists about which conservative management is preferable to support their patients.

### Trial status

Protocol version #2, October 01, 2019. Registered at Clinicaltrials.gov, identifier: NCT03576989. Currently recruiting. Trial start date was October 16, 2019. Anticipated recruitment end date is April 30, 2021.

### Study sponsor

Waldfriede Krankenhaus, Argentinische Allee 40, 14,163 Berlin, Berlin, Germany. Tel.: 004930818108201, Email: m.bock@waldfriede.de

## Supplementary information

**Additional file 1.**

**Additional file 2.** SPIRIT 2013 Checklist: Recommended items to address in a clinical trial protocol and related documents

## Data Availability

Information and data sets gathered as a result of this trial will be available from the corresponding author upon reasonable request. Results and findings of the study will be released through publications in the scientific literature and conference presentations.

## Abbreviations

AE: Adverse event
CMC-1: Carpometacarpal 1
DASH: Disabilities of the Arm, Shoulder, and Hand questionnaire
IP: Interphalangeal
MCP-1: Metacarpophalangeal 1
MHT: Multimodal hand therapy
OAT: Osteoarthritis of the trapeziometacarpal joint
SF-36: Short Form 36
SUR: Study of Medical Ultrasound for Rhizarthrosis
TUS: Therapeutic ultrasound
VAS: Visual analogue scale
