# Supplementary material for: Study of Medical Ultrasound for Rhizarthrosis (SUR): study protocol for a randomized controlled single-center pilot-trial
Source: Trials. 2020 Jun 1;21:450. doi: 10.1186/s13063-020-04375-2 (PMC7268749; doi:10.1186/s13063-020-04375-2)
Supplement: Supplementary file 1 — Additional file 1. [file 13063_2020_4375_MOESM1_ESM.docx]

Study of medical ultrasound for rhizarthrosis (SUR)

-Patienteneinverständniserklärung-

Version1.2

Hiermit erkläre ich,

Frau/Herr___________________________________________________________________

geboren am__________________________________________________________________

wohnhaft in_________________________________________________________________

(Telefon)____________________________________________________________________

(Email)_____________________________________________________________________

dass ich durch Frau/Herrn Dr. med ______________________________________________

aus dem Krankenhaus Waldfriede e.V., Hand- und Fußchirurgie, Argentinische Allee 40, 14163 Berlin, über das Wesen, die Bedeutung, Tragweite und Risiken dieser wissenschaftlichen Studie informiert wurde und ausreichend Gelegenheit hatte, meine Fragen hierzu in einem Gespräch mit dem Studienarzt zu klären.

Ich habe insbesondere die mir vorgelegte Patienteninformation Version 1.2 verstanden und eine Ausfertigung derselben und dieser Einwilligungserklärung erhalten. Mir ist bekannt, dass ich meine Einwilligung jederzeit ohne Angabe von Gründen und ohne nachteilige Folgen für mich zurückziehen und einer Weiterverarbeitung meiner Daten jederzeit widersprechen und ihre Löschung bzw. Vernichtung verlangen kann.

Ich bin bereit an der wissenschaftlichen Studie „Study of medical ultrasound for rhizarthrosis“ teilzunehmen.

**Einwilligungserklärung zu Datenverarbeitung**

Ich erkläre mich damit einverstanden, dass im Rahmen dieser Studie erhobene Daten über meine Gesundheit verschlüsselt und auf elektronischen Datenträgern aufgezeichnet verarbeitet und die anonymisierten Studienergebnisse veröffentlicht werden.

Ort, Datum

___________________________________________________________________________

Unterschrift des/der Patienten/Patientin Unterschrift Studienarzt
